# Blood Flow of the Acral Finger Arterioles in Patients With Type 2 Diabetes by Quality Doppler Profiles

**DOI:** 10.1007/s12013-013-9561-4

**Published:** 2013-03-23

**Authors:** Tao Zhang, Liang-hua Xia, Yan-yan Bian, Bo Feng, Chao Wang, Fan-xia Meng, Yu-hui Zhang, Ming Chen

**Affiliations:** 1 Division of Ultrasound, Hui-nan Community Health Service Center, Pudong New District, Shanghai, 201301 People’s Republic of China; 2 Division of Noninvasive Cardiac Function, Heart Center, Shanghai East Hospital, Tongji Medical School, Tongji University, 150 Jimo Road, Pu Dong, Shanghai, 200120 People’s Republic of China

**Keywords:** Ultrasound, Diabetes mellitus, type 2, Complications, Finger, Arterioles

## Abstract

Patients with diabetes mellitus exhibit peripheral arterioles lesions that is associated with reduced blood flow. Here, we intended to assess the acral arterioles lesion in patients with type 2 diabetes based on the rate of blood flow by multigate spectral Doppler ultrasonography. Fifty-two patients with type 2 DM were divided into two groups. Group 1 included 13 men and 12 women with an average age of 60.60 ± 14.03 years and a duration of type 2 diabetes for 2.44 ± 1.50 years. Group 2 included 17 men and 11 women with an average age of 64.25 ± 10.84 years and type 2 diabetes for 12.57 ± 6.26 years. Age-matched control subjects (*n* = 52) were recruited (30 men and 22 woman, mean age of 61.19 ± 10.38 years). A multigate spectral Doppler algorithm was applied to the acral finger of the thumb of the right hand to test the arteriole diameter and hemodynamic parameters, including diameter of the acral finger arterioles (*D*), area of the blood flow profile of the acral finger arterioles (*A*_max_) and hemodynamic parameters. Patients with diabetes exhibited a significant reduction in the arteriole diameter (1.63 ± 0.18 and 1.57 ± 0.22 mm, respectively, *P* < 0.001 for both) compared to control subjects (2.09 ± 0.17 mm). *A*_max_ were significantly reduced in patients with diabetes (61.35 ± 10.66 mm^2^/s for group 1 and 46.50 ± 6.59 mm^2^/s for group 2, *P* < 0.001 for both) compared to that in control subjects (77.93 ± 12.37 mm^2^/s). Furthermore, a significant difference in Amax was found between group 1 and group 2 (*P* < 0.001). The vascular resistance index (*RI*) was significantly higher in both patient groups 0.58 ± 0.06 for group 1 (*P* < 0.001) and 0.64 ± 0.07 for group 2 (*P* < 0.001) than that in control subjects (0.48 ± 0.04). The *RI* value of the acral finger arterioles differed significantly between group 1 and group 2 (*P* < 0.01). Diabetic patients exhibited a weak blood flow in the acral finger arterioles. The multigate spectral Doppler technology can be used to test blood flow in the acral finger arterioles and provide hemodynamic data for systematic analyses of the peripheral arteriole lesions in diabetes.

## Introduction

Diabetes mellitus (DM) is a common disease, causing increasing public health problems [1]. It is well-known that diabetes often leads to peripheral arteriole disorders in association with microvascular complications [2–5]. Occasionally, diabetes increases the risk of microvascular disorders without any clinical signs and symptoms [6, 7]. Identification of early vascular lesions in the arterioles of the extremities in patients with DM are critical for prompt treatment, especially for observing the reversible effect of medication during the early stage of the DM [8, 9]. Although ultrasonography is one of most popular approach for detection of blood flow in small blood vessels noninvasively [10], ultrasonic image blurring is one of the major problems for detecting arteriole lesions in patients with DM. Recently, a quality Doppler profiles (QDP) technology has been developed and successfully used for testing cerebral venous outflow in patients with multiple sclerosis [11]. This technology is sensitive to detect blood flow with low signal intensity [11–13]. We hypothesize that weak blood flow in acral finger arterioles could be monitored as a useful physical finding of vascular lesions in the arterioles of the in patients with DM. In this investigation, we tested hemodynamic parameters of the acral finger arterioles to evaluated the peripheral arteriole lesions in patients with type 2 diabetes by the quality Doppler profiles technology.

## Methods

### Diabetic and Control Subjects

Fifty-three patients with type 2 DM based on 1999 diagnostic criteria for DM (30 men and 23 woman, age 62.53 ± 12.46 years) were selected from August, 2011 to April, 2012 in Department of Endocrinology in Shanghai East Hospital. Diagnosis of type 2 DM in all patients was defined according to the guidelines of the American Diabetes Association [14]. The patients with ketoacidosis, hyperosmolar nonketotic diabetic coma, acute infection, acral ischemic gangrene, or autoimmune disease were excluded. All of 53 patients were recruited in the present study and were classified into two groups according to duration of the type 2 DM. Group 1 consisted of 25 patients with type 2 DM including 13 males and 12 females, mean age 60.60 ± 14.03 years, with FBG (fasting blood-glucose) 9.52 ± 3.72 mmol/L and glycated hemoglobin (HBALC) was 9.74 % ± 2.19 %, whose duration of type 2 DM ≤5 years (2.44 ± 1.50 years in average). Group 2 consisted of 28 patients with type 2 DM including 17 males and 11 females, mean age 64.25 ± 10.84 years, with FBG in 8.94 ± 4.79 mmol/L and HBALC in 9.99 ± 2.68 %, who were diagnosed on the basis of the duration of the type 2 DM > 5 years (12.57 ± 6.26 years in average). Eleven were smokers (≈4 cigarettes/d) in both diabetic groups (6 in Group 1 and 5 in Group 2). The study protocol was approved by the local Ethics Committee of Shanghai East Hospital and all participants gave their informed consent.

Fifty-two healthy subjects were recruited (30 men and 22 woman, age 61.19 ± 10.38 years). These subjects were free from hypertension and MD. Eight were smokers (≈ 4 cigarettes/d) in the control. None of the subjects had evidence of macrovascular or cardiovascular disease as determined by history, physical examination, and ECG, and none were taking any medication.

### Ultrasonography

The ultrasonic system (ESAOTE-Biosoud Mylab 25 color Doppler Imaging Meter, Genoa, Italy) equipped with a two-dimensional color and pulse Doppler function was used with a linear array broad bandwidth electron transducer (LA332 Vascular probe) at a frequency of 10 MHz, which permits detection of flow at a minimum velocity about 1 cm/s. Color scale (the pulse repetition frequency) was set at 1.4 kHz to avoid aliasing. Insonation angle compensation for color Doppler was kept at 65 degrees or smaller. The color gain was adjusted to insure the satisfied color filling inside the vessel.

#### Flow Index Measured by Ultrasonography (Image Acquisition and Data Processing)

**Fig. 1:**
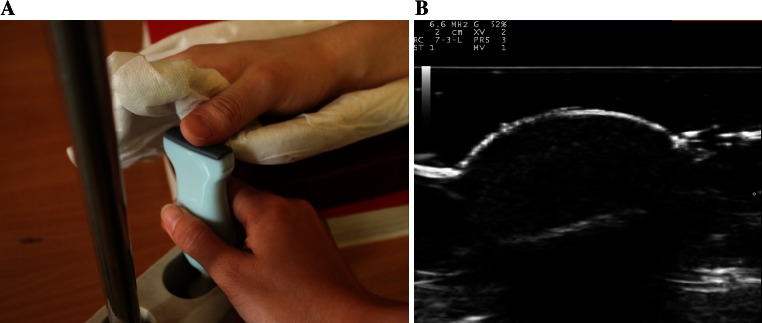
**a** A finger plaster cast made with gypsum for fixing thumb of the right hand of examinee and the transducer was put in the transducer holder with surface of transducer facing up to thumb of the right hand of examinee. The thumb of the right hand of examinee was gently facing down and putting on the surface of the transducer which was perpendicular to the axis of the thumb. The transducer inside the transducer holder was shifting by operator. **b** The transducer should touch the surface of the thumb softly to keep the arch of the surface of the thumb on two-dimensional image of ultrasound. The long-axis of the acral arterioles was applied to keep the angulation between sound beam and blood flow ≤60°

The thumb of the right hand was chosen for ultrasound examination. Patients were instructed to lie flat on an examination couch with palms facing down. A finger plaster cast made with gypsum was used to hold the thumb of the right hand (Fig. 1a). The thumb of the right hand of examinee was placed gently on the surface of the transducer which was perpendicular to the axis of the finger and the longitudinal depiction of the acral finger arterioles was accomplished during examination (Fig. 1b).

**Fig. 2:**
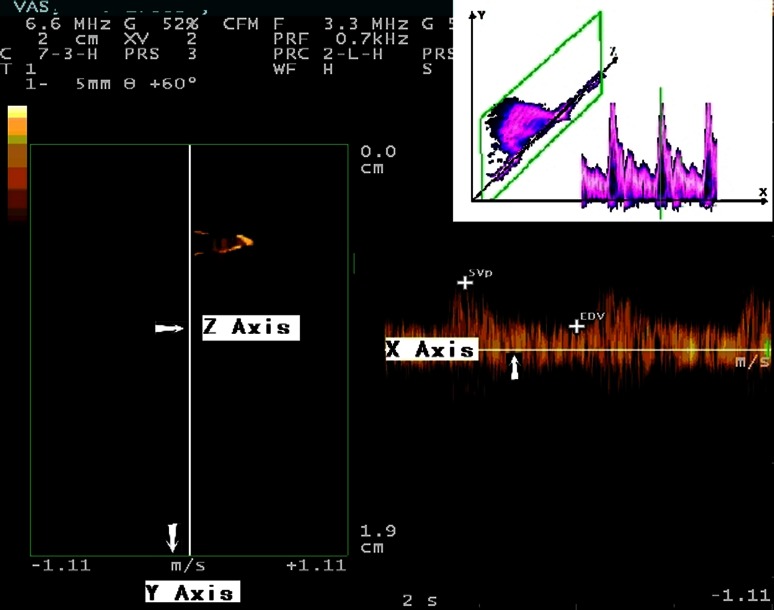
Flow profile of the arterioles of the finger from QDP spectrum with the Directional Algorithm (DIR Software). On the QDP spectrum, the *X*-axis represents time (in second), *Y*-axis means velocity of blood flow (in cm/s) and *Z*-axis is the multigated depths of the lumen of the arterioles (in mm)

A color Doppler mode was activated while the pulsation of the acral finger arterioles was seen with the depth of 2.0 cm and blood flow velocity was 7 cm/sec. Then, the short-axis image of the acral finger arterioles in color Doppler and two-dimensional ultrasound was displayed. Because the acral finger arterioles are the transverse arterial branches between two proper palmar digital arteries in the thumb of the right hand, the transducer was put along the long-axis of the acral finger arterioles to keep the angulation between sound beam and blood flow ≤60 and angle correction was made in all patients. Multigate spectral Doppler, a technique for processing the echo signals backscattered from multiple depths along the ultrasound beam, was applied to measure the blood vessel dimension and produce flow profiles. The Doppler spectrum and flow spectrum in the acral finger arterioles were constructed and analyzed using the QDP algorithm. When the pulse motion of the wall of the acral finger arterioles was found on the two-dimensional image, a Doppler sampling cursor (20 mm in length) containing 256 sampling gates was placed over the acral finger arterioles. Then, the QDP flow of the image was started with the gain function properly adjusted to clearly display blood flow waves in the acral finger arterioles (Fig. 2). The color Pulse Repeat Frequency (PRF) was adjusted to visualize the signals of the acral finger arterioles.

### Doppler Measurements

A MATALAB algorithm was designed to measure blood flow profiles and calculate the rate of blood flow in the acral finger arterioles. Using the computational algorithm, we measured the diameter of the arterioles and the area of flow waveform. It was designed on the basis of image segmentation of digital image processing [15]. The proposed diameter and area of flow wave of the acral finger arterioles on the QDP spectrum were measured by the following steps: (1) importing the image of QDP spectrum, (2) selecting the region of interest, (3) identifying the edge of flow profiles on the QDP spectrum by utilizing the image segmentation technique, (4) Contouring the flow spectrum on the imported image, (5) calculating the area of flow wave during systole on the QDP spectrum. Blood flow in the acral finger arterioles was directly determined using the coronal section, while the short-axis section of the acral finger arterioles was visualized by B-mode ultrasonography. Flow signals of the acral finger arterioles could be detected clearly on the spectrum of QDP. A typical spectral waveform is shown in Fig. 2.

**Fig. 3:**
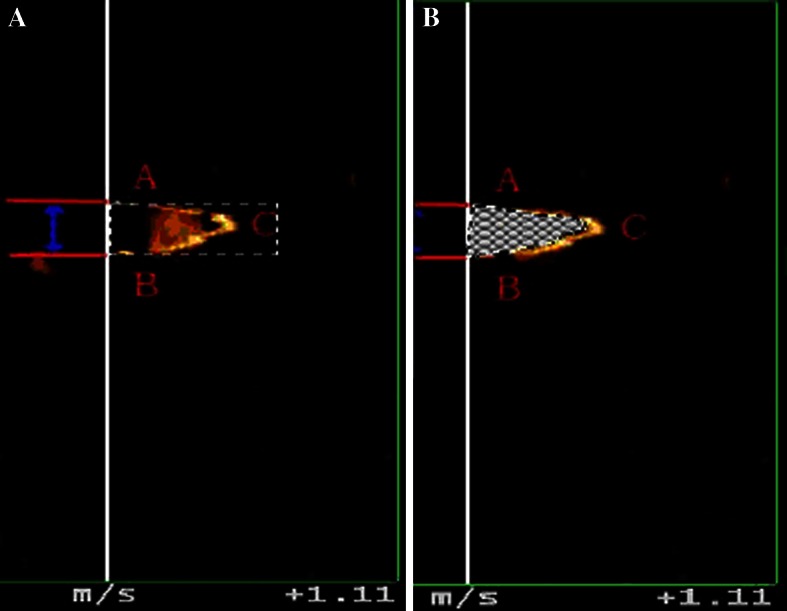
On the QDP spectrum, *Y*-axis represents velocity of blood flow and *Z*-axis means the multigated depths of the lumen of the arterioles. *Left panel*: The distance between the onset point at ascending part of flow wave (**a**) and ending point at descending branch (**b**) of it on the spectral profile along the *Z*-axis was corresponding the predicting size of the acral finger arterioles (*D*, in mm) (**a**). Area of the blood flow profile of the acral finger arterioles was calculated as *A*_max_ (in mm^2^/s) by a software designed in our laboratory (**b**)

All flow signals intercepted by ultrasound beam could be sampled by application of multigate spectral Doppler from the QDP algorithm. The size of the acral finger arteries was measured from the flow spectrum using QDP technique with a 256 sample gating system [11]. While the 256 sample gating system was applied to the center of the acral finger arterioles, the diameter of the acral finger arterioles (*D*, in mm) could be measured from the flow spectrum (Fig. 3). On the blood flow profile of the acral arterioles, *Y*-axis represents velocity of flow of the arterioles (in mm^2^/s) and *Z*-axis means the multigated depths of the lumen of the arterioles (in mm). The area of the maximal blood flow profile of the acral finger arterioles from the flow spectrum (*A*_max_ in mm^2^/s) was measured from the QDP spectrum using the software established in our laboratory (Fig. 3).

We established an experimental program to measure the area of the acral arterioles on QDP spectrum in our laboratory. It is designed on the basis of image segmentation of digital image processing. In color gradient analyses for RGB images, Di Zenzo has established a method for gradient-based edge detection [16]. The gradient image $F_{\theta } (x,y)$, which is the vector function extension of gradient, was the key of color edge detection [17].

1 $$ F_{\theta } (x,y) = \left\{ {\frac{1}{2}\left[ {(g_{xx} + g_{yy} ) + (g_{xx} - g_{yy} )\cos 2\theta + 2g_{xy} \sin 2\theta } \right]} \right\}^{1/2} $$

2 $$ \theta \left( {x,y} \right) = \frac{1}{2}\arctan \left[ {\frac{{2g_{xy} }}{{(g_{xx} - g_{yy} )}}} \right] $$

where $\theta \left( {x,y} \right)$ provides the direction of the maximum rate of change in RGB image, and *g*_*xx*_, *g*_*yy*_, and *g*_*xy*_ represent the dot product of vectors u and v, defined as below:

$$ g_{xx} = {\mathbf{u}} \cdot {\mathbf{u}} = {\mathbf{u}}^{T} {\mathbf{u}} = \left| {\frac{\partial R}{\partial x}} \right|^{2} + \left| {\frac{\partial G}{\partial x}} \right|^{2} + \left| {\frac{\partial B}{\partial x}} \right|^{2} $$

3 $$ g_{yy} = {\mathbf{v}} \cdot {\mathbf{v}} = {\mathbf{v}}^{T} {\mathbf{v}} = \left| {\frac{\partial R}{\partial y}} \right|^{2} + \left| {\frac{\partial G}{\partial y}} \right|^{2} + \left| {\frac{\partial B}{\partial y}} \right|^{2} $$

$$ g_{xy} = {\mathbf{u}} \cdot {\mathbf{v}} = {\mathbf{u}}^{T} {\mathbf{v}} = \frac{\partial R}{\partial x}\frac{\partial R}{\partial y} + \frac{\partial G}{\partial x}\frac{\partial G}{\partial y} + \frac{\partial B}{\partial x}\frac{\partial B}{\partial y} $$

where vector u and v are expressed as below:

$$ {\mathbf{u}} = \frac{\partial R}{\partial x}{\mathbf{r}} + \frac{\partial G}{\partial x}{\mathbf{g}} + \frac{\partial B}{\partial x}{\mathbf{b}} $$

4 $$ {\mathbf{v}} = \frac{\partial R}{\partial y}{\mathbf{r}} + \frac{\partial G}{\partial y}{\mathbf{g}} + \frac{\partial B}{\partial y}{\mathbf{b}} $$

where vector r, g, and b represent the unit vectors in a RGB color space along the R, G, and B axis. It provided quantitative data by calculating the pixel spacing and proportion in a segmented arterioles image. Then, the data of area inside the lumen of arterioles were collected and computed (Fig. 3).

A flow velocity-depth plot of the acral finger arterioles was constructed based on three cardiac cycles. All procedures were performed by the same experienced ultrasonographist. Peak systolic velocity (PSV) and end diastolic velocity (EDV) were measured from the Doppler spectrum by the QDP technology. The vascular resistance index (RI) of the acral finger arterioles was calculated based on the formula RI = PSV − EDV/PSV.

### Statistical Analysis

Means and standard deviations were calculated for each measured parameter. Each value was expressed as mean ± standard error. The unpaired Student’s *t* test was used for normally distributed continuous variables. Differences were considered statistically significant when *P* value was less than 0.05.

## Results

**Fig. 4:**
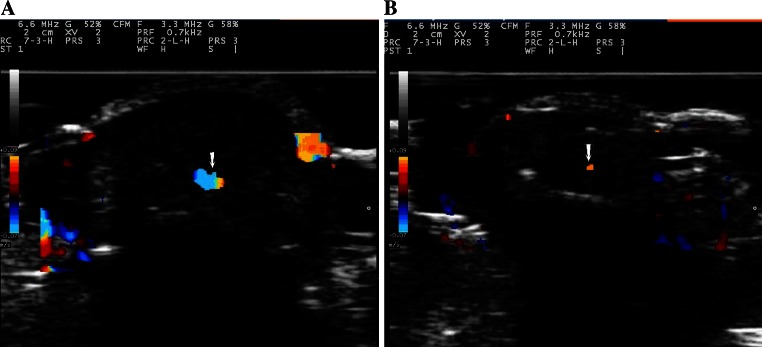
**a** Shows rich pulsed flow signals (*white arrow*) in *bright blue color* were observed inside the acral finger arterioles in a healthy subject. **b** Shows *poor red color* (*white arrow*) was seen inside the acral finger arterioles in a patient with type 2 DM

Abundant blood flow from pulse flow signals were displayed for the acral finger arterioles in all 52 healthy subjects (Fig. 4a). However, no patient with type 2 DM had a full filling feature in arteriole image as shown in normal subjects. Only scattered flow signals were seen in the center of acral finger arterioles in patients with type 2 DM by two-dimensional and color Doppler ultrasonography (Fig. 4b).

**Fig. 5:**
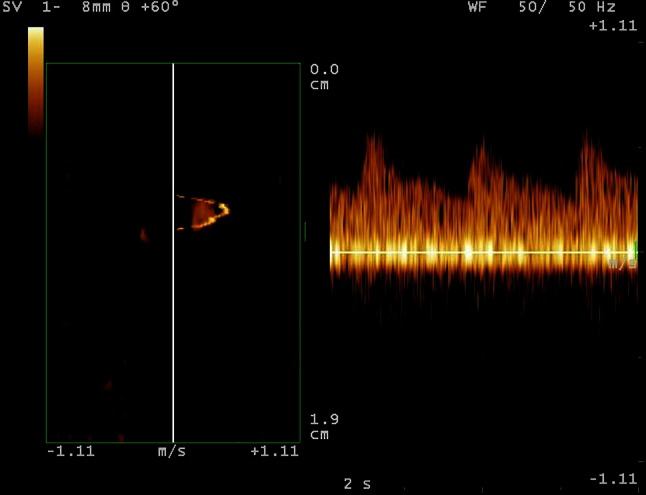
Blood flow profile in the acral finger arterioles obtained by the algorithms of multigate spectral Doppler in a healthy subject. The spectral flow profile in the acral finger arterioles is displayed in *left panel* and corresponding flow wave of pulsed Doppler is shown in *right panel*

**Fig. 6:**
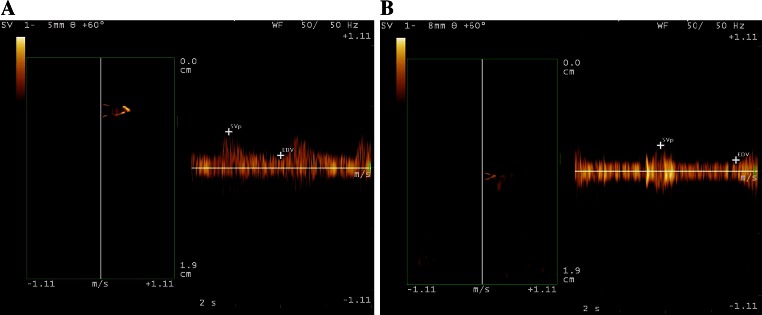
**a** Shows blood flow profile in the acral finger arterioles obtained by multigated spectral Doppler using QDP technique in a patient with type 2 DM in Group 1 (duration of DM ≤5 years and **b** shows that in a patient in Group 2 (duration of DM >5 years), in which both the *D* and *A*_max_ measured from the flow profile of the acral finger arterioles in a patient in Group 2 were smaller than that of in a patient in Group 1

The spectral flow signals of the acral finger arterioles were detected in all 52 control subjects, but only 48 patients with type 2 DM showed the spectral flow signals (90.57 %). Even in 5 patients with type 2 DM (9.61 %) in Group 2, no flow signals were detected in the acral finger arterioles in 5 patients with type 2 diabetes. Compared with conventional pulsed Doppler or color Doppler, blood flow could be detected clearly on the flow spectrum using the QDP technology in all cases in this study. Doppler flow of the acral finger arterioles was detected in healthy subjects (Fig. 5) and patients with type 2 DM in both Group 1 and Group 2 using the QDP algorithm (Fig. 6).

Doppler flow data of the acral finger arterioles were obtained in 52 healthy persons and 53 patients with type 2 DM, including Group 1 (*n* = 25) and Group 2 (*n* = 28) using the QDP technology. Table 1 shows the mean values with standard deviations of diameter of the acral finger arterioles measured from healthy subjects and the two patient groups. There were significant differences in D and A_max_ from the systolic blood flow curve of the acral finger arterioles between the patient groups and the control (all *P* < 0.001, Table 1).

**Table 1:** Flow characteristics of the acral finger arterioles in patients with type 2 diabetes by quality Doppler profiles

	*n*	D (mm)	A_max_ (mm^2^/s)
Control	52	2.09 ± 0.17	77.93 ± 12.37
Group 1 (≤ 5 years)	25	1.63 ± 0.18^*^	61.35 ± 10.66^*^
Group 2 (> 5 years)	28	1.57 ± 0.22^**#**^	46.50 ± 6.59^**#▲**^

Data were presented as mean ± SD

*D* diameter of the acral finger arteriole, *A*_*max*_ maximal area of the flow profile of the acral finger arterioles, *Group 1* patient group with duration of type 2 diabetes ≤5 years, *Group 2* patient group with duration of type 2 diabetes >5 years

*P < 0.001: Group 1 vs Group of control; # *P* < 0.001: Group 2 vs Group of control; ^▲^ *P* < 0.001: Group 1 vs Group 2

Compared with that in the control, the diameter of the acral finger arterioles was reduced (2.09 ± 0.17 mm in the control vs 1.63 ± 0.18 mm in group 1 and 1.57 ± 0.22 mm in group 2, all *P* < 0.001) and *A*_max_ of the acral finger arterioles was decreased (61.35 ± 10.66 mm^2^/s in Group 1 and 46.50 ± 6.59 in Group 2 vs 77.93 ± 12.37 mm^2^/s in the control, all *P* < 0.001) in patient groups. Furthermore, no significant difference in the arteriole diameter between Group 1 and Group 2 was found (*P* > 0.05). However, there was a significant difference between Group 1 and Group 2 in *A*_max_ of the acral finger arterioles (*P* < 0.001).

Table 2 summarizes Doppler Flow characteristics of the acral finger arterioles in patients with type 2 Diabetes by the quality Doppler profiles technology. As the duration of DM increased, the PSV decreased in the group 2 patients compared with the control subjects (*P* < 0.01), but no significant difference in PSV was found between group 1 and the control (*P* > 0.05) and between group 1 and group 2 (*P* > 0.05) (Table 2). EDV were significantly lower in both patient groups than that in control subjects (all *P* < 0.001), but no significant difference in EDV was found between group 1 and group 2 (*P* > 0.05) (Table 2). The RI was significantly higher in both patient groups than that in control subjects (all *P* < 0.001). The RI value of the acral finger arterioles also differed significantly between group 1 and group 2 (*P* < 0.001) (Table 2).

**Table 2:** Doppler Flow characteristics of the acral finger arterioles in patients with type 2 Diabetes by quality Doppler profiles

	*N*	PSV (cm/sec)	EDV (cm/sec)	RI
Control	52	39.73 ± 14.19	20.59 ± 7.70	0.48 ± 0.04
Group 1 (≤5 years)	25	33.62 ± 12.12	13.70 ± 4.81^*^	0.58 ± 0.06^*^
Group 2 (>5 years)	28	31.83 ± 9.90^**##**^	11.34 ± 4.24^**#**^	0.64 ± 0.07^**#**▲▲^

Data were presented as mean ± SD

*PSV* peak systolic velocity, *EDV* end diastolic velocity, *RI* vascular resistance index, *Group 1* patient group with duration of type 2 diabetes ≤5 years, *Group 2* patient group with duration of type 2 diabetes >5 years

*P < 0.001: Group 1 versus Group of control; **P < 0.01 Group 1 vs Group of control; ^#^ *P* < 0.001: Group 2 vs Group of control. ^##^ *P* < 0.01;^▲^ *P* < 0.001: Group 1 vs Group 2; ^▲▲^ *P* < 0.01: Group 1 vs Group 2

## Discussion

This is the first report for the quantification of blood flow in the acral finger arterioles by the QDP technique, which enables us to demonstrate the blood flow of the acral finger arterioles and detect lesions in finger arterioles. DM is a common disorder with a range of serious life-threatening complications, including microvessel lesions. Usually, blood flow in the radial arterioles or dorsal arterioles can be monitored by means of vascular ultrasonography. However, it is difficult to visualize blood flow in the lumen of finger arterioles by conventional pulse wave Doppler because the size of acral finger arterioles is smaller than that of radial artery or dorsal artery. Doppler flow signals in the finger arterioles normally are difficult to detect regular color Doppler techniques, because of the poor circulating blood flow in the finger arterioles, especially, in patients with type 2 diabetes [11]. Conventional color Doppler or two-dimensional ultrasonography is not suitable for identifying the acral finger arteries in patients with type 2 Diabetes. Therefore, we use a new QDP technique to assess the size of the finger arterioles in diabetic peripheral arterial disease. The QDP technique was also used to detect heamodynamic changes in the finger arterioles in the present study. The QDP technique has been established by combining power spectral intensities corresponding to all the depths along the ultrasound beam. Each intercepted point could be scanned simultaneously with greater sensitivity and ability for precisely localizing blood flow by the QDP algorithm [11, 12, 18]. Actually the diameter (*D*) of the acral finger arterioles in the present study is the transverse arterial branches between proper palmar digital arteries in the thumb. The long axis of the acral arterioles could be scanned by 256 gated sampling processes in multiple depths along the ultrasound beam from the transverse section of the thumb. If a multiple sampling cursor was scanning at the path of the ultrasound beam, the multiple depths of arterioles including their vessel walls and lumens are simultaneously intercepted by the ultrasound beam and the arteriole can be well displayed. Each of the 256 sampling processes contributes to the construction of the flow wave profiles on the flow spectrum and the diameter of the acral finger arterioles can be tested accurately.

We observed that the acral finger arterioles is small with rich blood flow in healthy subjects, but the diameter of the acral finger arteries was diminished in both diabetic groups (*P* < 0.001 both in Group 1 and Group 2). We measured the spectral A_max_ of blood flow in the acral finger arterioles obtained by the algorithms of multigate spectral Doppler in the present study. From the spectral flow curve of the acral finger arterioles, maximal flow profile in systole was chosen to be a hemodynamic reference through the acral finger arterioles. The flow spectrum is a velocity versus multigated depth plot representing an instantaneous flow analysis through the acral finger arterioles in real time. Hence, the *A*_max_ measured from the spectral flow wave, represents the distribution of blood flow in the arterioles of the finger. The *A*_max_ of the acral finger arterioles was decreased in both patient groups (*P* < 0.001) in comparison with that in the control group. Due to the proportional relationship between the intensity of power Doppler and the number of scatterers of the red blood cells [19–21], blood flow through the sampled blood vessel can be detected quantitatively by measuring the diameter and *A*_max_ by this QDP method. Although there was no difference in the D of the acral finger arterioles between patient group 1 and group 2, significant difference in the *A*_max_ of the acral finger arterioles was found between group 1 and group 2 (*P* < 0.001) in this investigation. As the duration of DM increased, the distribution of blood flow in the arterioles became lower.

Diminished blood flow in the acral finger arterioles might be related to microcirculatory disorders including alterations in the metabolism of tissues [21], remodeling of arterioles [22, 23], and high blood coagulation [24] in patients with type 2 DM. There is a growing evidence linking microvascular complications with cerebrovascular and cardiovascular disease in patients with type 2 diabetes. Many diabetic complications are mediated through the diabetic toxic effects on blood vessels, including increased circulating microparticles, in patients with type 2 DM [25] .

Furthermore, our results showed that the RI of the acral finger arterioles was significantly higher in both patient groups than that in control subjects (all *P* < 0.001). The RI value also increased in group 2 than that of group 1 (*P* < 0.01) significantly, indicating that the RI value became higher as the duration of DM increased, except for PSV and EDV. These results indicate that DM could lead to injury of microvessels through several pathological mechanisms, including endothelial dysfunction and autonomic nervous system disturbances, which are characterized by arteriosclerosis of small arterioles [26]. Patients in group 2 had lower PSV in the acral finger arterioles compared with that in the control subjects (*P* < 0.01), and both patient groups had decreased EDV than that in the control subjects (all *P* < 0.001). As the duration of DM increased, blood flow decreased and blood flow resistance increased, as observed in this investigation.

Similar hemodynamic alterations in the arterioles of the finger and toe in patients with DM were observed by Ma et al. [27] using an e-flow Doppler technique, which was developed from the power Doppler sonographic method. Although blood flow in the acral finger arterioles could be shown by the e-flow Doppler technique, the flow hemodynamic parameters (PSV and EDV) measured by the e-flow Doppler technique were collected in a conventional manner with only one sampling line or sampling point. In this study, we found that it was difficult to detect the flow signals of the acral finger arterioles with the regular Doppler spectra and obtain RI, PSV, and EDV in 5 patients with type 2 DM in the present study. However, using the multigate sampling method with a QDP algorithm, distinct flow profiles of the acral finger arterioles were found on the flow velocity-depth plot of the Doppler spectrum, and both PSV and EDV could be measured using the 256 sampling technique in patients with type 2 DM in the present study. QDP imaging had advantages in acquisition of the finger arterioles blood flow compared to the conventional Doppler method, even though no signals of color blood flow were seen on the two-dimensional image of the acral finger arterioles.

Abnormal vascular changes of the toe ends occur earlier and was more severe than those of the finger ends. In the present study, only the finger arterioles were tested in patients with DM. Finger arterioles could be a convenient window for monitoring early pathological changes noninvasively, which might be used routinely to detect diabetic peripheral arterial disease by estimating hemodynamic parameters.

## Limitations

Arteriole blood flow can be affected by small pressure of transducer on the finger surface during data acquisition. Therefore, the finger surface should not be pressed with the transducer holder. In this investigation, the size of aural finger arterioles could not be measured using other medical imaging modalities, such as CT or MRI. The measured D and A_max_ can reflect the instantaneous blood flow changes in the finger arterioles. The flow profiles by the QDP algorithm are not only composed of the velocity but also the scatterers of the red blood cells through the arterioles [18, 19]. Therefore, this potential limitation of flow profiles will not affect the result in our observation in patients with DM. The limitation of the time and spatial resolution was resolved by maximal flow profiling to catch the maximal pulsating flow in the finger arterioles using the QDP technique.

## Conclusion

The hemodynamic parameters of the acral finger arterioles in patients with type 2 DM could be detected by the QDP algorithm. The QDP technique used to detect hemodynamic insufficiency of the finger arterioles. Both anatomic and pathological change in the acral finger arterioles can be readily identified in diabetic patients from flow profile from the QDP spectrum. Weak blood flow existed in the acral finger arterioles in patients with DM and become worse as disease progresses.
